# Core competencies in the science and practice of knowledge translation: description of a Canadian strategic training initiative

**DOI:** 10.1186/1748-5908-6-127

**Published:** 2011-12-09

**Authors:** Sharon E Straus, Melissa Brouwers, David Johnson, John N Lavis, France Légaré, Sumit R Majumdar, K Ann McKibbon, Anne E Sales, Dawn Stacey, Gail Klein, Jeremy Grimshaw

**Affiliations:** 1 Li Ka Shing Knowledge Institute, St Michael's Hospital, 30 Bond Street, Toronto, ON, M5B 1W8, Canada; 2 Department of Oncology, McMaster University, Juravinski Hospital, G Wing, Room 207, 711 Concession Street, Hamilton, ON, L8V 1C3, Canada; 3 Alberta Children's Hospital, Department of Pediatrics, Emergency Medicine, 2888 Shaganappi Trail NW, Calgary, AB, T3B 6A8, Canada; 4 Centre for Health Economics and Policy Analysis, McMaster University, Program in Policy Decision-Making, 1280 Main St. West,CRL-209, Hamilton, ON, L8S 4K1, Canada; 5 Department of Family Medicine, Université Laval, Québec, PQ, G1K 7P4, Canada; 6 General Internal Medicine, 2F1.24 WMC, University of Alberta, Edmonton, AB, T6G 2B7, Canada; 7 Centre for eHealth, McMaster University, Health Information Research Unit, 1280 Main Street West, CRL-132, Hamilton, ON, L8S 4K1, Canada; 8 VA Inpatient Evaluation Center (IPEC), HSRD (152), VA Ann Arbor Healthcare System, PO Box 130170, Ann Arbor, MI, 48105, USA; 9 University of Ottawa, School of Nursing, 451 Smyth Road, Rm 1480F, Ottawa, ON, K1H 8M5, Canada; 10 Clinical Epidemiology Program, Ottawa Hospital Research Institute, The Ottawa Hospital - General Campus, 501 Smyth Road, Box 711, Ottawa, ON, K1H 8L6, Canada

## Abstract

**Background:**

Globally, healthcare systems are attempting to optimize quality of care. This challenge has resulted in the development of implementation science or knowledge translation (KT) and the resulting need to build capacity in both the science and practice of KT.

**Findings:**

We are attempting to meet these challenges through the creation of a national training initiative in KT. We have identified core competencies in this field and have developed a series of educational courses and materials for three training streams. We report the outline for this approach and the progress to date.

**Conclusions:**

We have prepared a strategy to develop, implement, and evaluate a national training initiative to build capacity in the science and practice of KT. Ultimately through this initiative, we hope to meet the capacity demand for KT researchers and practitioners in Canada that will lead to improved care and a strengthened healthcare system.

## Introduction

Globally, health systems fail to optimally use evidence with resulting inefficiencies and reduced quantity and quality of life [1-6]. Recognition of this challenge has created interest in knowledge translation (KT) or implementation science. This growing emphasis on KT has led to the establishment of an interdisciplinary field of KT research and the need to enhance capacity in KT to meet the demand.

Similar to the situation in other countries, we have a shortage of people trained in the science and practice of KT in Canada. To respond to this challenge, we are developing a national training initiative (funded by the Canadian Institutes of Health Research, or CIHR, from 2009 through 2015) including colleagues from eight universities. It was established to enhance capacity in the science and practice of KT by:

1. Providing innovative training centres and laboratories for trainees from various research disciplines (including clinical epidemiology, health services research, social sciences, engineering, and health informatics, and from different professions including medicine, nursing, engineering, and psychology) to develop skills in KT and KT research.

2. Linking trainees and mentors to collaboratively advance the science and practice of KT.

3. Partnering with other national and international research groups to promote KT research and training of well-rounded trainees across a range of settings, and clinical and health system issues.

In our literature search to identify KT training initiatives, we were unable to identify any national KT training strategies that we could model. To develop our strategy, we considered the need to advance both the science and practice of KT and decided that to enhance capacity we should focus training on three streams: Stream 1 includes graduate (MSc and PhD) and advanced (postdoctoral) training in the science and practice of KT; Stream 2 includes training in the basic principles of the science and practice of KT for researchers from other areas such as basic science and health services research; and Stream 3 includes basic training in the practice of KT for any knowledge users interested in enhancing their knowledge and skills for practicing KT.

## The KT Training Streams

Several educational theories and principles can guide the development of an educational program. Common elements that form the basis of our program include the assessment of learning needs, facilitation of social interaction between learners, and provision of opportunities to practice new skills [7]. People have different learning styles, and inclusion of a range of teaching techniques are used to meet these needs including active learning through small group work, interactive discussions (seminars and asynchronous discussions), and brief didactic sessions [8]. Elements of cognitive learning theory influence the program development of Stream 1, particularly the use of mentorship to support learners [7]. Adult learning theory influences all streams, assuming that learners have acquired knowledge, are motivated to learning material relevant to their needs and are self-directed.

**Figure 1:**
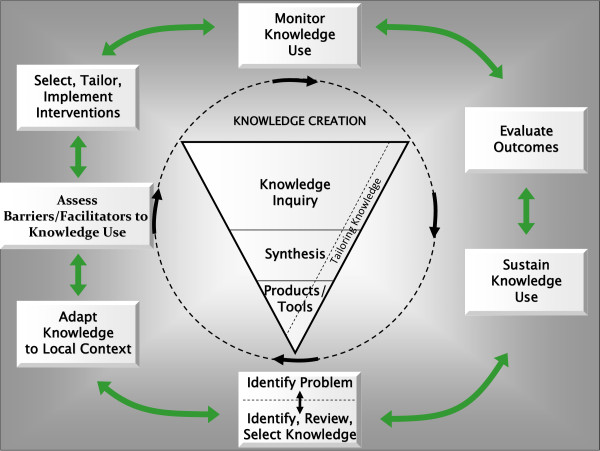
**Knowledge to Action Loop**.

Two frameworks guide our training curriculum: the Medical Research Council (MRC) Framework for Complex Interventions and the Knowledge to Action loop [9,10]. Our ultimate goal is to improve the quality of care through the development and evaluation of KT interventions in real world settings to provide practical guidance to healthcare stakeholders (including clinicians, patients, policy makers, and managers) about optimal KT strategies. The UK MRC Framework for Complex Interventions [9] extends from contextual assessment and development of the theoretical basis for an intervention through to development, evaluation and cost-effectiveness of an intervention, and to evaluation of its sustainability. This framework was used to identify the core competencies for Stream 1 trainees that are described below. The second framework that informs the training curriculum and development of the core competencies is the Knowledge to Action loop developed by Graham *et al*. [10] (Figure 1). It highlights processes relating to knowledge creation, distillation, and use. This framework may be particularly helpful to strategies targeting clinicians, patients, citizens, and managers, but may be less helpful for strategies targeting policy makers because many policy maker targeted interventions may focus on facilitating access to research in a timely fashion rather than supporting behavior change.

### Stream 1 training

This stream focuses on the science and practice of KT including skills in the research methods relevant to the phases of the MRC Framework. The practice of KT focuses on skill development in end-of-grant KT (disseminating research results and engaging stakeholders in using them) and integrated KT (collaborative activities that engage knowledge users to ensure that the research is relevant to and used by the knowledge users).

The core competencies for Stream 1 trainees are based on the MRC and KTA Frameworks and include: knowledge and understanding of models and theories of KT and KT research; capacity to conduct syntheses to address KT questions, specifically reviews of complex interventions which may include consideration of qualitative and quantitative research; capacity in multiple research methods including qualitative methods to examine the determinants of knowledge use across different settings and stakeholder groups; and capacity to design and evaluate the impact, effectiveness, and sustainability of KT strategies in different settings.

Overarching each of these competencies is the need to develop skills in engaging relevant stakeholders (including the public, healthcare providers, managers, and policy makers) to facilitate an integrated KT approach.

Each of these competencies (Table 1) is addressed through a variety of educational initiatives including modular courses, a national seminar series, an annual Summer Institute, yearly research meetings, and a research practicum if desired by the trainee.(Table 1) We are exploiting technology to ensure national accessibility of these educational activities.

**Table 1:** Core competencies for Stream 1 trainees and educational initiatives targeting these competencies

Educational Initiative	Core Competency			
	**Models and theories of KT and KT research**	**KT Synthesis**	**Research methods in KT**	**Developing, evaluating, sustaining KT Interventions**

Modular courses in systematic reviews, pragmatic randomized trials, end-of-grant KT	X	X	X	

Monthly e-seminar series	X	X	X	X

Summer Institute	X	X	X	X

Mentorship	X	X	X	X

Courses in systematic reviews of complex interventions and in pragmatic trials of KT interventions are available online and in person (Table 2). Similarly, we have developed courses in end-of-grant KT to help trainees as they prepare grants.

**Table 2:** Summary of Courses Available in 2011

Course Name	Audience	Date
Introduction to Evidence Based Medicine	Trainees, researchers, physicians, knowledge users, *et al*.	Winter 2011

End-of-grant KT	Trainees, researchers, physicians, knowledge users, *et al*.	Summer 2011

Introduction to Systematic Reviews	STIHR trainees, other trainees, researchers, physicians, knowledge users, *et al*.	Winter 2011 - STIHR trainees Fall 2011 - Others

Pragmatic KT Trials	STIHR trainees, other trainees, researchers, physicians, knowledge users, *et al*.	Summer 2011

A monthly e-seminar series (topics in Table 3) focuses on KT research methodology. Webcasts of these seminars are available on our KT Canada website [11]. A quarterly 'Research Operations' e-seminar series is being offered in 2011 for students focusing on writing grants; reviewing grants; preparing presentations, grants, ethics submissions, and manuscripts; and retrieving relevant literature and discussing ethical issues in KT research and project management. One of the outputs from the initial student seminar was to develop an online series of interviews with KT experts who outline their career paths and what factors influenced their career choice. This online series is available on our program website.(http://ktclearinghouse.ca/).

**Table 3:** Topics for KT Canada Monthly Seminar Series

Year	Topic	Presenter(s)
2008	Implementation Science - a Letter from the UK	Dr Martin Eccles

2009	Supporting Research Use by Health System Managers and Policymakers	Dr John Lavis

	A Shared Decision Making Approach to Knowledge Transfer and Exchange	Dr France Légaré

	Ethical Challenges in Knowledge Translation Research	Dr Charles Weijer

	Patient Decision Aids as a Knowledge Translation Strategy: Opportunities and Barriers	Dr Annette O'Connor

	Cluster RCT Comparing Three Methods of Implementing Practice Guidelines for Children with Croup	Dr. David Johnson

	Collaborative Journal Clubs: Can Bottom Up Implementation Work?	Prof Paul Glasziou

	How We Use the Language of KT, and Its Challenges: Time for Changes?	Dr Ann McKibbon

	The Influence of Social Networks on KT in Long Term Care Facilities	Dr Anne Sales

	A Cognitive Perspective of Knowledge Translation	Dr Jamie Brehaut

2010	Advancements in Development, Reporting and Evaluation of Clinical Practice Guidelines: Introducing AGREE II	Dr Melissa Brouwers

	Translating Nutrition Research into Practice: A Look at Novel Strategies to Improve Evidence-Based Diet-Related Decisions	Dr Sophie Desroches

	Making Guidelines Easier to Follow: Bridging Best Evidence and a Clear Message	Dr Onil Bhattacharyya and Dr Monika Kastner (Post-Doctoral Fellow)

	Lost in Translation: How I Found the Way	Dr Ian Graham

	Storytelling as a KT Strategy in Child Health: Croup as an Illustrative Example	Drs Lisa Hartling, Terry Klassen and Shannon Scott

	KT Trials Must Overcome both Poor Practitioner and Patient Performance	Dr Brian Haynes

	Sustainability of KT Innovations	Dr Barbara Davies

	Studying the Use of Research Knowledge in Public Bureaucracies	Dr Mathieu Ouimet

2011	Policy Makers and Researchers as Partners in Knowledge Mobilization	Ms Nancy Reynolds and Dr Suzanne Tough

	Required Versus Inspired Partnerships: Report on a Survey of Researchers and Knowledge-Users Holding Integrated KT Grants	Ms Jacqueline Tetroe

	KT Research at Toronto Rehabilitation Institute: Using Psychological Theory to Improve the Implementation of a Hand Hygiene Intervention	Dr Veronique Boscart and Dr Susan Jaglal

	Supporting evidence based practice through information technologies	Dr Diane Doran

	Evidence-based self-management: translation of knowledge into a self-management tool for patients with urinary incontinence	Dr Jayna Holroyd-Leduc

	Translating knowledge to support e-health implementation in healthcare: A multidimensional approach	Dr Marie-Pierre Gagnon

	Applying to Integrated Knowledge Translation Funding Opportunities at CIHR: Tips for Success	Mr Ryan McCarthy and Mr Adrian Mota

	Leading for Quality and Safety	Dr Deborah White

Graduate students are invited to participate in the annual KT Summer Institute, which focuses on a different theme each year and addressing one or more of the core KT competencies, including developing KT interventions and targeting them to different stakeholder groups. To date, we have held three Summer Institutes with involvement of 90 trainees (Table 4). These Institutes include didactic and active learning with small group work focused on an assigned KT project and exposure to mentors. Trainees also present their research in progress during facilitated poster sessions to gain skills in presentation. To date, trainees have been involved with three publications [12]. They have also been involved with preparation of collaborative, multi-site grants (Tables 5, 6 and 7). Similarly, trainees have worked together on education modules and presentations; *e.g*., two trainees presented at a recent Cochrane meeting to outline the methodological challenges in doing reviews of qualitative literature and subsequently submitted a grant on this topic. Trainees have developed collaborations in other projects including a community of practice [11] that has received funding to host meetings to develop this community.

**Table 4:** Participants attending KT Canada Summer Institutes

Year	Theme	# of Attendees	Trainee Level (%)	Trainee Demographics (%)	Trainee Language (%)
2009	Exploring the Knowledge to Action Framework	30	Master's: 7 PhD: 80 Post Doc: 13	Alberta: 10 British Columbia: 7 Manitoba: 3 Nova Scotia: 3 Ontario: 53 Quebec: 17 Saskatchewan: 7	English: 83 French: 17

2010	Developing KT Interventions	29	Master's: 10 PhD: 62 Post Doc: 28	Alberta: 17 Ontario: 59 Quebec: 24	English: 72 French: 28

2011	Integrating the Science and Practice of KT	31	Master's: 13 PhD: 58 Post Doc: 29	Alberta: 6.4 British Columbia: 3.2 Manitoba: 3.2 Ontario: 58 Pennsylvania: 3.2 Quebec: 26	English: 77 French: 23

**Table 5:** Funded Trainees

Level of Training	# of Funded Trainees
Master's	0

PhD	9

Post-Doctoral Fellow	5

**Table 6:** Demographics of Funded Trainees

University	# of Funded Trainees
McMaster University	2

Queen's University	1

University of Alberta	3

University of British Colombia	1

Université Laval	2

University of Ottawa/The Ottawa Hospital Research Institute	2

University of Toronto	3

**Table 7:** Trainee Collaborations

Initiative	Description	Planned or Actual Output
KT Trainee Collaborative	Trainees from past summer institutes came together to create a network of KT trainees	1. Two CIHR meeting, planning and dissemination grants for meetings in Winnipeg (Mar 2010) and Toronto (Apr 2011) 2. Poster presentation at KT 10 meeting in Halifax Jun 2010 (Colquhoun *et al*.) 3. Poster presentation at Family Medicine Forum meeting in Vancouver Oct 2010 (Urquhart *et al*.) 4. Poster presentation at RTNA meeting in Edmonton Oct 2011 (Richmond *et al*.) 5. Publication: Cornelissen E, Urquhart R, Chan V *et al*. Creating a knowledge translation collaborative: from conceptualization to lessons learned in the first year. Implementation Science 2011; 6:98 http://ktclearinghouse.ca/kttc/

Summer Institute Publications	Trainees from the CIHR sponsored KT summer institute (2008) and the 2009 KT Canada summer institute (SI) have published two meeting reports in Implementation Science	1. Kho M, Estey E, Deforge R *et al*. Riding the knowledge translation roundabout: lessons learned from the Canadian Institutes of Health Research Summer Institute in knowledge translation. Implementation Science 2009;4:33 2. Leung BY, Catallo C, Riediger ND, Cahill NE, and Kastner M. The trainees' plan on developing and end-of-grant knowledge translation plan. Implementation Science 2010;5:78 3. Bhogal S, Menon A, Bath B *et al*. Using problem-based case studies to learn about knowledge translation interventions: An inside perspective. Journal of Continuing Education in Health Professionals. *In Press*

CIHR Knowledge Synthesis Grant	Trainees and investigators across multiple sites collaborated on a synthesis grant to strengthen their understanding of the concept of replication and identify a useful framework to guide replication research in KT	1. Grant awarded from CIHR for $96,352 to investigators at OHRI (Grimshaw, Brehaut, Moher), SMH (Straus), McMaster (McKibbon), U of Alberta (Sales) and trainees at OHRI (Curran, Vachon) 2. Review of the social science, education, business, and health literature using multiple search strategies to identify relevant literature and a theory analysis to identify and define major concepts and elements. 3. Model case approach to examine the extent to which replication research is evident in knowledge translation research. 4. Invite knowledge users and stakeholders to participate in development of recommendations for replication research practice for researchers, policy makers, funders and journal editors.

RCT Protocol	Two trainees from the 2010 SI along with investigators from the OHRI are working on a KT Intervention to improve the long-term use of evidence-based medication in patients diagnosed with cardiovascular disease. This is currently in the planning phase.	1. Collaboration between trainees from McMaster (Schwalm) and U of Toronto (Ivers), investigators at OHRI (Grimshaw, Taljaard) and at McMaster (Natarajan) 2. Provincial baseline assessment of discontinuation rates of CV medications in patients over 65 years old post cath 3. Small feasibility trial in STEMI patients 4. LHIN-wide randomized controlled clinical trails

Collaboration with University of Newcastle, Australia	Prof Robert Sanson-Fisher, a member of KT Canada's scientific advisory board, attended the 2010 summer institute and offered to host trainees in Australia, a testimony to the strength of our trainees	This collaboration is currently in the planning phase, and the KT Canada steering committee is exploring other possible opportunities with members of the scientific advisory and other interested international KT leaders

Graduate students are expected to do a KT-focused thesis that may include supervision from mentors at more than one participating institution. Graduate students and fellows from disciplines including clinical epidemiology, informatics, nursing, medicine, psychology, health policy, business, computer science, and engineering amongst others are brought together through this program and encouraged to work together. More than 60 faculty members from across Canada are involved with the training initiative and are available to provide mentorship. All of the faculty hold CIHR grants as principal investigators for KT research projects. New trainees meet with the Program Director to explore their interests and goals. This discussion is used to identify potential mentors if the trainee does not have one. During the Summer Institute, opportunities to meet with the potential mentors are available. These opportunities include a 'speed mentoring' session on the first day that invites the trainees to meet with multiple potential mentors during 15-minute sessions. These sessions are focused on identifying if there is interest in exploring a mentoring relationship. Trainees also have the opportunity to meet with other mentees who work with that mentor to determine if it might be a good fit. Longer meeting sessions are then available on the second day of the Summer Institute to facilitate mentorship. Our mentorship approach is based on the results of three systematic reviews of mentorship and a large qualitative study of mentorship that we completed [13-15]. For example, we found that assigning mentors can lead to a superficial or artificial relationship, and instead it is preferred that mentees are given a list of potential mentors and provided with opportunities to meet with each.

Each Stream 1 trainee is expected to complete an annual learning profile and objectives, which are reviewed with their primary mentors. A summary is reviewed with the trainee and the Program Director during a yearly interview to discuss progress and concerns. The mentors and Program Director work together to create sustainable momentum in supporting KT research careers, to provide trainees with skills for lifelong success and collaboration, and to foster an attitude of lifelong learning.

Eligible applicants for Stream 1 include trainees enrolled in a graduate program or fellowship with a focus on KT. We encourage applicants from across Canada. Each application is independently reviewed by two KT researchers using a standard scale used by the CIHR review panels. Candidates with the highest scores are offered a stipend and the opportunity to participate in the training activities described above. Details of the application process are provided in Additional File 1. We open the Summer Institute to trainees who do not receive Stream 1 funding and use a similar application process to that described above.

### Stream 2 training

Training in the principles of KT is available for researchers and trainees from other fields using distance-learning technologies. The core competencies for this stream include training in both end-of-grant KT and integrated KT. A one-day, in-person session on end-of-grant KT is available and we are currently working to make this available online. We have also implemented a modular, integrated KT course, reflecting the knowledge to action loop. This course has been offered to a number of groups; for example, we have developed a partnership with the Michael Smith Foundation for Health Research (a funding agency responsible for healthcare research in British Columbia) and the Vancouver Coastal Health Research Institute (an organization involving seven hospitals and various research programs) to provide a number of courses and have recently submitted a grant to evaluate impact over two years. Modules on end-of-grant KT and integrated KT are also available for online completion (http://ktclearinghouse.ca/). The KT handbook, entitled 'Knowledge Translation in Healthcare' [16] provides the basis for courses developed for this stream.

### Stream 3 training

Stream 3 targets decision makers (including clinicians, healthcare managers, and policy makers) who want to know more about what KT is and how to do it in their own setting. Two courses are available to focus on the core competency of how to implement a KT project in their organisation: a brief introductory session providing an overview of KT and a modular course that including the basics of KT, and an opportunity for participants to apply them directly to a project in their own setting. This latter course has been held on two occasions including colleagues from 16 teams. Topics include: what is KT, how can I do KT in my own setting, and how do I implement, monitor and sustain KT strategies in my own setting?

## Faculty Development

Mentorship is a key component of this initiative and while the key mentors have extensive mentorship expertise, ongoing faculty development will be available for mentors and Stream 1 trainees. A mentorship program and tools have been developed based on our research including several systematic reviews on this topic [13-15]. Mentorship tools (*e.g*., individual development plans, interactive case discussions) have been used at sessions including the Summer Institutes and will be evaluated in this training initiative. We are also completing a series of interviews with expert mentors to provide strategies and tactics for effective mentorship and these are available online. Our work is aimed to give trainees the skills and professional training that will allow them to become leaders in KT and KT research and to mentor future generations of researchers.

## Evaluation of the KT Canada Training Initiative

Ensuring that this training initiative meets its objectives will require a multicomponent process. Core measures will include: number of trainees in each of the three streams (and their discipline); numbers of publications, research presentations, grants, honours, programs developed and implemented by trainees, impact of research, and engagement with relevant stakeholders; and number of KT researchers recruited and retained. Summative evaluation will include surveying participants from all three streams about their perceptions and experiences with this initiative and its effect on employment, position, and their practice of KT; and, surveying team members about their experiences and perceptions of the initiative. In a formative evaluation strategy, each year a sample of trainees from each of the three streams will be invited to participate in a semi-structured interview to explore their experiences with the initiative, their perceptions of effective/ineffective components and to propose revisions to the training program. The results of these evaluations will be used to continuously refine and improve this initiative.

## Costs of the Program

This program is funded by the CIHR ($1.7 million over six years) and two-thirds of the funding must be used for student stipends. We have obtained additional grant support to provide activities such as the Summer Institute. We are actively seeking partnerships to sustain and grow the program. For example, we have a partnership with the British Medical Journal to fund fellows interested in KT and health informatics and are exploring similar partnerships with other interested stakeholders including provincial funding agencies.

Strengths of this initiative include unique linkages with relevant stakeholder audiences and the tremendous breadth and depth of expertise of the members in KT and KT research. These linkages will facilitate sustainability of the training. Furthermore, sustainability will be enhanced through offering courses to our collaborators from decision-maker organisations as well as to our colleagues from other training and research initiatives. Ultimately through this initiative, we hope to meet the capacity demand for KT researchers and practitioners in Canada that will lead to improved care and a strengthened healthcare system.

## Competing interests

Drs. Grimshaw, Légaré, Lavis, and Brouwers are members of the Editorial Board of Implementation Science; Dr. Sales is an Associate Editor of Implementation Science.

## Authors' contributions

SES wrote manuscript. MB provided input and revisions. DJ provided input and revisions. JNL provided input and revisions. FL provided input and revisions. SRM provided input and revisions. KAM provided input and revisions. AES provided input and revisions. DS provided input and revisions. GK provided input, revisions and tables. JG provided input and revisions. All authors read and approved the final manuscript.

## Supplementary Material

Additional file 1 **STIHR Application Process**. Here we describe the process by which students apply to the different training opportunities. Included are: the application requirements, the instructions for reference letters, and the review criteria. Click here for file

## References

[B1] McGlynn E Asch S Adams J Keesey J Hicks J DeCristofaro A Kerr EA The quality of health care delivered to adults in the United States N Engl J Med 2003 348 2635 45 10.1056/NEJMsa022615 12826639

[B2] Kiesler DJ Auerbach SM Optimal matches of patient preferences for information, decision-making and interpersonal behavior: evidence, models and interventions Patient Educ Couns 2006 61 3 319 341 10.1016/j.pec.2005.08.002 16368220

[B3] O'Connor AM Bennett C Stacey D Barry MJ Col NF Eden KB Entwistle V Fiset V Holmes-Rovner M Khangura S Llewellyn-Thomas H Rovner DR Do patient decision aids meet effectiveness criteria of the International Patient Decision Aid Standards Collaboration? A systematic review and meta-analysis Med Decis Making 2007 27 5 554 74 10.1177/0272989X07307319 17873255

[B4] Shah BR Mamdani M Jaakkimainen L Hux JE Risk modification for diabetic patients. Are other risk factors treated as diligently as glycemia? Can J Clin Pharmacol 2004 11 2 e239 e244 15557673

[B5] Pimlott NJ Hux JE Wilson LM Kahan M Li C Rosser WW Educating physicians to reduce benzodiazepine use by elderly patients: a randomized controlled trial CMAJ 2003 168 7 835 839 12668540 PMC151988

[B6] Kennedy J Quan H Ghali WA Feasby TE Variations in rates of appropriate and inappropriate carotid endarterectomy for stroke prevention in 4 Canadian provinces CMAJ 2004 171 5 455 459 10.1503/cmaj.1040170 15337725 PMC514641

[B7] Hutchinson A Estabrooks C Straus SE, Tetroe J, Graham I Theories of KT: Educational theories Knowledge Translation in Health Care 2009 Oxford: Wiley Blackwell

[B8] Lewis AP Bolden KJ General practitioners and their learning styles J R Coll Gen Pract 1989 39 187 199 2560001 PMC1711996

[B9] Craig P Dieppe P Macintyre S Michie S Nazareth I Petticrew M Medical Research Council Guidance Developing and evaluating complex interventions: the new MRC guidance BMJ 2008 337 979 83 10.1136/bmj.a979 PMC2769032 18824488

[B10] Graham ID Logan J Harrison MB Straus SE Tetroe J Caswell W Robinson N Lost in knowledge translation: time for a map? J Contin Educ Health Prof 2006 26 1 13 24 10.1002/chp.47 16557505

[B11] http://ktclearinghouse.ca Accessed June 2010

[B12] Kho M Estey E DeForge R Mak L Bell BL Riding the knowledge translation roundabout: lessons learned from the CIHR Summer Institute in Knowledge Translation Implementation Science 2009 4 33 10.1186/1748-5908-4-33 19523216 PMC2700786

[B13] Sambunjak D Straus SE Marusic A The impact of mentorship: systematic review JAMA 2006 296 1103 15 10.1001/jama.296.9.1103 16954490

[B14] Straus SE Chatur F Taylor M Issues in the mentor-mentee relationship in academic medicine: A qualitative study Academic Medicine 2009 84 135 9 10.1097/ACM.0b013e31819301ab 19116493

[B15] Straus SE Graham ID Lockyer J on behalf of the Alberta Mentorship Working Group Development of a mentorship strategy. A KT Case Study J Contin Educ Health Prof 2008 28 117 22 10.1002/chp.179 18712802

[B16] Straus SE, Tetroe J, Graham I Knowledge Translation in Health Care. 2009 Oxford: Wiley Blackwell

