# Supplementary material for: Core competencies in the science and practice of knowledge translation: description of a Canadian strategic training initiative
Source: Implement Sci. 2011 Dec 9;6:127. doi: 10.1186/1748-5908-6-127 (PMC3292943; doi:10.1186/1748-5908-6-127)
Supplement: Additional file 1 — STIHR Application Process. Here we describe the process by which students apply to the different training opportunities. Included are: the application requirements, the instructions for reference letters, and the review criteria. [file 1748-5908-6-127-S1.DOC]

Additional File 1 – STIHR Application Process

Training opportunities are advertised through the KT listserv, CIHR’s KT newsletters and Twitter. Interested trainees are invited to submit an application online at <http://applications.ktclearinghouse.ca/>. The application consists of the following information:

1. Application form: includes name, academic institution, program of study, level of study, supervisor name and position
2. Essay question: Please provide a description of your interest in Knowledge Translation and Knowledge Translation research and describe how this Training Program would facilitate your career goals. (500 words max)
3. CIHR Common CV, including publication and presentation lists
4. Post secondary transcripts

Trainees are also asked to have 2 reference letters and assessment forms sent directly to the training program manager. Instructions are provided for the referees:

Instructions to the Referee: Thank you for agreeing to provide a letter of support for the candidate. The information that you provide about the candidate is crucial for the Selection Committee in their evaluation of the applicant. We ask that you complete the assessment form about the applicant in comparison to other individuals at the same level of training and experience with whom you have worked. In addition to the completion of the assessment form, we ask that you provide a letter of reference in support of the candidate’s application which includes how long you have known the applicant, in what capacity you have known the candidate, and an assessment of the applicant with particular reference to their performance in a clinical or research training, and their potential to perform in a research training program.

Name of Applicant:___________________________

Name of Referee:____________________________

Title of Referee:______________________________

Contact details of Referee:______________________

| Item | Exceptional (top 5%) | Excellent (top 10%) | Very Good (top 20%) | Good (top 30%) | Acceptable (below 30%) | Not able to assess |
| --- | --- | --- | --- | --- | --- | --- |
| Sense of Responsibility |  |  |  |  |  |  |
| Motivation |  |  |  |  |  |  |
| Organisational skills |  |  |  |  |  |  |
| Judgment/critical thinking |  |  |  |  |  |  |
| Intellectual ability |  |  |  |  |  |  |
| Research skills |  |  |  |  |  |  |
| Originality |  |  |  |  |  |  |
| Collaborative skills |  |  |  |  |  |  |
| Interpersonal skills |  |  |  |  |  |  |
| Teaching skills |  |  |  |  |  |  |
| Ability to complete projects |  |  |  |  |  |  |

Each application is reviewed by two members of the training program’s planning committee using a developed set of review criteria:

**Application Review Criteria**

**Eligibility**

Applicants must meet the following eligibility criteria:

1. Be Canadian citizens, have landed immigrant status, or be an international student enrolled at a Canadian university
2. Be enrolled in a graduate program or hold a post-doctoral fellowship at a Canadian university, or be in the process of applying to a graduate program or post-doctoral fellowship at a Canadian university.

**Review Criteria**

1. Preference will be given to current PhD students and postdoctoral/clinical fellows
2. Preference will be given to applicants who hold a national or provincial research award (e.g., CIHR, SSHRC, MSFHR, AHFMR, FRSQ, NSHRF, MHRC, CHSRF etc.)
3. Demonstrated research interests and (where relevant) thesis topics aligned with knowledge translation or knowledge translation research in research areas

**Scoring System** Scores out of 60

**1. Preferential Criteria 10 points total**

Degree: 5 points for PhD or Postdoctoral student/fellows

2 points for Masters Student

Site: 5 points for being at a preferred training site and/or in receipt of an externally funded award. To be considered a preferred training site, the supervisor must be a KT Canada member.

0 points otherwise

**2. Research Interests 15 points total**

*High score (12 -15 points)*

The research topic and/or students’ interest is of direct relevance to knowledge translation and/or knowledge translation research. Highest priority will be given to applicants whose research interests are directly associated with knowledge translation and knowledge translation research (design, implementation, evaluation of knowledge translation interventions, assessments of knowledge translation impact of policies in health and other sectors, evidence synthesis methods, assessment of sustainability of interventions) in the area(s) of health services and policy research and/or population and public health research. Both qualitative and quantitative methodologies will be accepted.

*Medium Score (8-11 points*)

Somewhat relevant to knowledge translation and knowledge translation research and framed in a way that is linked to this, e.g., needs assessment leading to an intervention; examining barriers to behaviour change or system change to promote best patient care; research on capacity building to promote uptake of interventions; research on interventions with clinical populations. Those applicants expressing an interest in Knowledge translation and Knowledge translation research but neither is the main focus of their **current** or previous research activities will also be considered.

*Low Score (1-7 points)*

Of little relevance to knowledge translation or knowledge translation research, and little interest in either.

**3. Self Description and Career Goals 15 points total**

High points for articulation, sophistication, curiosity, originality, interests in keeping with problem-focused, solution-driven, knowledge translation research or intervention, good match of learning style with that of the Training program.

**4. CIHR Common CV 5 points total**

High points for publications and presentations at conferences

**5. Post-Secondary Transcripts 5 points total**

High points for high GPA.

**6. Reference Letters 10 points total**

High score (8-10) for ratings in the exceptional and excellent range, medium score (5-7) for ratings of very good and good and low score (1-4) for acceptable ratings.

| **Review Criteria** | **Details** | **Points** | **Score** | |
| --- | --- | --- | --- | --- |
| **Preferential Criteria** | | **10** |  |  |
| Degree | - 5 points for PhD or Postdoctoral student - 2 points for Masters Student |  |  | **/5** |
| Site | - 5 points for being at a preferred training site and/or in receipt of an externally funded award - 0 points of otherwise |  |  | **/5** |
| **Research Interests** | The research topic and/or students’ interest is of direct relevance to knowledge translation and/or knowledge translation research  ***High score: 12 -15 points***  ***Medium Score: 8-11 points***  ***Low Score: 1-7 points*** | **15** |  | **/15** |
| **Self Description and Career Goals** | High points for articulation, sophistication, curiosity, originality, interests in keeping with problem-focused, solution-driven, knowledge translation research or intervention, good match of learning style with that of the Training Program. | **15** |  | **/15** |
| **CIHR Common CV** | High points for publications and presentations at conferences | **5** |  | **/5** |
| **Post-Secondary Transcripts** | High points for high GPA. | **5** |  | **/5** |
| **Reference Letters** | High score (8-10) for ratings in the exceptional and excellent range, medium score (5-7) for ratings of very good and good and low score (1-4) for acceptable ratings. | **10** |  | **/10** |
| **Total** | | |  | **/60** |
